# I Feel! Therefore, I Am from Pain to Consciousness in DOC Patients

**DOI:** 10.3390/ijms241411825

**Published:** 2023-07-23

**Authors:** Francesco Riganello, Paolo Tonin, Andrea Soddu

**Affiliations:** 1 Research in Advanced Neurorehabilitation, S. Anna Institute, 88900 Crotone, Italy; 2 Physics, and Astronomy Department, Western Institute for Neuroscience, University of Western Ontario, London, ON N6A 3K7, Canada

**Keywords:** pain, nociception, disorders of consciousness, consciousness

## Abstract

Pain assessment and management in patients with disorders of consciousness (DOC) is a challenging and important aspect of care, with implications for detecting consciousness and promoting recovery. This narrative review explores the role of pain in consciousness, the challenges of pain assessment, pharmacological treatment in DOC, and the implications of pain assessment when detecting changes in consciousness. The review discusses the Nociception Coma Scale and its revised version, which are behavioral scales used to assess pain in DOC patients, and the challenges and controversies surrounding the appropriate pharmacological treatment of pain in these patients. Moreover, we highlight recent evidence suggesting that an accurate pain assessment may predict changes in the level of consciousness in unresponsive wakefulness syndrome/vegetative state patients, underscoring the importance of ongoing pain management in these patients.

## 1. Introduction

Pain is a universal response to harmful stimuli, and is a fundamental aspect of the evolutionary process in living organisms [1,2]. Our limited understanding of pain’s development restricts our comprehension of its biology, and little is known about the relationships between pain-related states in animals and humans or the evolution of the processes required for these states. Pain is a complex, multidimensional experience essential for survival and adaptation, serving as a vital communication system between the body and the brain [3]. It alerts an organism to potential threats and prompts it to take appropriate action [4]. Investigations of cellular mechanisms and behavioral responses related to nociceptor activation, tissue injury, inflammation, and the environmental context of these responses are starting to reveal the evolution of mechanisms and behaviors important for pain [2]. Consequently, pain has emerged as a universal response across diverse living beings, facilitating their ability to thrive and propagate their genetic material across generations [5,6].

## 2. Nociception Is Not Pain

The term “noxious” originates from the Latin noxius (“hurtful, injurious”), noxa (“hurt, injury”), and nocere (“to hurt, injure”). The significance is applicable to both physical and emotional distress.

Nociception refers to the peripheral neurophysiological pathways within the sensory nervous system that detect and relay harmful stimuli (thermal, mechanical, chemical, or electrical) from the body to the spinal cord [3]. These biological signals mediate between external events and an organism’s internal state, prompting reflexive behavior to protect the organism. This response can occur before pain and potentially without perception, making it an evolutionary trait found across species [2].

On the other hand, pain is a more conscious experience, often resulting from perceiving nociceptive information from external or internal sources [3]. The brain processes this information, making us consciously experience the stimulus as painful, thus prompting us to consider its various characteristics. This leads to complex behavioral expressions. Individual differences play a significant role in pain perception and reaction. Moreover, there is a complex relationship between these individual differences, cognitive processes, and the modulation of pain-related brain responses. Seminowics and Davis [7] used functional MRI (fMRI) to examine how a demanding cognitive task (Stroop) affects pain-related brain activity and vice versa. The study found that cognitive strategies can modulate pain-related brain regions, and different coping strategies may be related to behavioral subgroups in chronic pain patients.

Understanding the influence of cognitive strategies on pain-related brain activity sheds light on the complex interplay between mental and physical processes, and it has implications for chronic pain management.

It was suggested that effective prevention and management approaches to chronic pain should consider the biological, psychological, socio-demographic, and lifestyle factors that influence and result from pain [8].

Phenomena like placebo analgesia or distraction-induced pain relief underscore the strong impact that cognitive functions and learning systems have on our perception of pain [9].

Studying women’s fears and attitudes to childbirth showed that it might influence the maternity care they receive and birth outcomes. Hines and colleagues [10] demonstrated that in a clustering classification, the women belonging to the “fearful” cluster had a negative birth experience compared with the women clustered as “self-determined”. The comprehension of women’s perspectives and their fear levels was helpful in assisting midwives and doctors customize their communication with women.

Pain research suggests that how individuals think and behave can influence how they perceive and respond to pain, and that different subjects might react very differently to the same stimulus due to diverse sociocultural factors, cognitive beliefs, and expectations.

All these factors raise philosophical and ethical questions about our ability to truly comprehend another person’s qualitative pain experience since the variability in pain perception and expression is influenced by numerous factors, including the person’s physical state, cognitive thoughts, and cultural upbringing.

## 3. From Nociception to Pain

In the context of human or mammalian pain, several molecules and their receptors are implicated in nociception and pain perception. For example, Acid-sensing ion channels (ASICs) and membrane-bound receptors that mediate nociceptive responses to acids play a role in acid-induced pain perception in mammals [11,12]. They are expressed in mammalian mechanosensory neurons and are activated by a low pH or protons [11].

Transient receptor potential (TRP) channels are another group of molecules involved in pain perception [13]. They are expressed in mammalian nociceptors, they underpin thermal (heat) nociception, they are responsible for detecting and transducing noxious thermal stimuli into electrical signals, and they play a role in temperature-related pain perception in mammals [13].

Nociceptors are sensory neurons that respond to potentially damaging stimuli by sending signals to the spinal cord and brain [14].

Sherrington was a pioneer in defining the term “nociceptor” [15], which is the neural system that detects harmful stimuli. These harmful stimuli can include temperature extremes, mechanical shocks, and chemical threats that can potentially damage tissue.

Nociceptors are specialized nerve endings that primarily respond to harmful stimuli that may cause tissue damage. A significant number of nociceptors are polymodal, meaning they respond to a mix of these stimulus types. However, studies in rodents [16,17] have sparked a debate about the extent of polymodality, which may depend, in part, on whether the nociceptors innervate superficial or deep targets [18]. A subset of nociceptors, known as mechanically insensitive or silent nociceptors, do not respond to any modalities in their natural state but only react to stimuli when inflammation is present. This demonstrates the ability of nociceptors to become more sensitive in disease states [19].

In mammals, nociceptors are classified into two types: small myelinated A-delta fibers and smaller unmyelinated C fibers [20] (Table 1). These nociceptors are present in both peripheral tissues, [20] deep tissues, or viscera [21,22], and they have lower conduction velocity than A fibers. Electrophysiological and anatomical studies have characterized different types of nociceptors in mammals, including C fibers that are sensitive to mechanical stimuli, thermal stimuli, or both [23]. Some C fibers, known as “silent” C fibers, are only activated by heat when sensitized [23]. Cutaneous fibers in mammals are primarily nociceptive, whereas a smaller proportion serves touch and pressure functions [24,25]. This indicates that the mammalian nociception system can discriminate between different types of noxious stimuli to generate appropriate responses and avoid tissue damage.

Aδ mechanonociceptors are fast-conducting fibers [26] that are believed to transmit initial pain signals [27]. These fibers have higher mechanical thresholds than touch fibers, and some are also responsive to cold temperatures. Some Aδ fibers innervate the epithelial layer and are activated by noxious stimuli. A distinct group of Aβ-nociceptors, which are heavily myelinated, are specialized in detecting high-threshold mechanical stimuli. These neurons have faster conduction velocities compared with Aδ fibers, and they are not highly responsive to low-intensity brush stimuli [28].

C fibers, on the other hand, are thought to underlie long-lasting pain perception. Aδ stimulation is associated with pricking pain, whereas C fiber activation is associated with pressing and dull pain [29]. Approximately one-third of C fibers in mammals are polymodal, meaning they respond to mechanical, thermal, and chemical stimuli [30].

Nociceptors play a crucial role in encoding stimulus modality, intensity, and duration. They transmit this information to the central nervous system and facilitate nocifensive withdrawal responses [3].

Nociceptors extend their central axons from the tissues they innervate to second-order neurons located in either the trigeminal subnucleus caudalis or the dorsal horn of the spinal cord. In the spinal cord, A fibers establish connections with neurons in laminae I, II, and V, whereas C fibers form synapses in laminae I and II before undergoing modulation and/or transmission to the brain [14,26]

In healthy systems, the process of detecting harmful stimuli starts with afferent activation. When the signal is strong enough, it results in a complex pain perception that includes both discriminative and affective components.

## 4. Nociception, Inflammatory Process, and Pain

Inflammation and its relation to pain are interconnected processes within the body. The immune system naturally responds to injuries, infections, or irritants through inflammation, and pain often emerges as a prominent symptom of this inflammatory response [31,32]. Inflammation is an evolutionarily conserved process, with its primary role being to protect the host by eliminating pathogens and combating bacteria, viruses, toxins, and infections. Moreover, inflammation plays a pivotal role in facilitating tissue repair and promoting overall recovery, thus aiding in restoring normal bodily functions [33].

Inflammation can manifest in both acute and chronic forms. Acute inflammation is the immediate response to sudden bodily damage or injury, such as a cut or sprained ankle. It is a short-lived response designed to heal the damage and restore balance within the body [34,35]. In contrast, chronic inflammation occurs when the body continues to dispatch inflammatory cells even when there is no external threat [36]. Inflammatory mediators, like cytokines, can directly stimulate nerve endings, thus leading to pain perception. The release of these inflammatory substances can also sensitize nerve fibers, making them more responsive to pain signals [31,37,38].

The processes of nociception, inflammation, and pain are intricately intertwined in the perception of, and response to, harmful stimuli. Nociceptors, when activated, release neuropeptides and neurotransmitters that contribute to inflammation, which subsequently triggers the recruitment of immune cells and the release of inflammatory mediators [39]. The ensuing inflammatory response sensitizes nociceptors, leading to pain perception. G protein-coupled receptors (GPCRs) play a critical role in modulating nociceptor signaling and the inflammatory response, thereby influencing pain processing [14,40].

## 5. Key Modulators of Pain Perception: GPCR Ligands

Receptors, typically protein molecules found on the cell surface, recognize and bind to specific ligands such as hormones, neurotransmitters, and drugs, subsequently triggering physiological responses. G protein-coupled receptors (GPCRs), the largest protein family encoded by the human genome, play an integral role in cellular signal transduction [41,42]. They participate in many physiological functions and serve as targets for diverse therapeutic drugs, including those utilized for pain management [41,43].

Cannabinoid receptors, a part of the GPCR family, significantly impact pain perception. The endocannabinoid system encompasses two primary receptors (CB1 and CB2), their endogenous ligands—endocannabinoids, and related enzymes—which are all extensively distributed within pain circuits. This broad distribution makes the endocannabinoid system a potential therapeutic target for pain management. Endocannabinoids can modulate nociceptive signaling, whereas exogenous cannabinoids (such as those found in *Cannabis* spp.) have exhibited analgesic effects in both animal and human studies [44].

Adenosine receptors, another class of GPCRs, are also involved in pain modulation. Activating these receptors, specifically the A1 and A2A subtypes, can suppress the release of excitatory neurotransmitters and inhibit pain signal transmission, thereby providing another pathway for pain control [45,46].

The interaction of GPCRs with opioids is especially noteworthy in the context of pain management. Opioids such as morphine and tramadol bind to GPCRs, reducing pain signals and altering emotional responses to pain [47,48].

Interestingly, there is an overlap between the opioid and cannabinoid systems in pain modulation. Both systems, connected through GPCRs, share similar intracellular signaling pathways, suggesting a synergistic effect between cannabinoids and opioids for pain relief [49].

In addition to GPCR ligands, other ligands like the NR4A can also influence pain perception. The NR4A family comprises orphan nuclear receptors that are rapidly induced by various stressors. Despite the absence of identified endogenous ligands, these receptors are critical for maintaining cellular homeostasis and mitigating disease states, including inflammation and inflammatory diseases [50,51], which, in turn, can influence pain perception.

## 6. Pain and Homeostasis

Pain plays a crucial role in maintaining overall health and well-being by promoting homeostasis, which helps the body maintain a stable internal environment in response to changing external conditions [52]. When an organism experiences pain, homeostasis is disrupted, creating an unpleasant state. In response to injury or inflammation, pain signals trigger physiological responses such as immune activation, tissue repair, and endorphin release [53]. These responses work together to promote healing, mitigate further damage, and reduce the experience of pain.

Acute pain is a defensive mechanism that signals an immediate and active threat, but this definition does not fully encompass chronic pain, which persists even in the absence of an active threat or cause. Chronic pain is now recognized as a disease by itself [54].

Homeostatic dysregulation is a cornerstone of diverse neural disorders, particularly chronic pain, which is a major health problem worldwide.

## 7. Pain and Role of the Insula

The insula seems to have a basic role in homeostatic regulation. Craig described a somatotropic order in pain-ascending pathways, terminating at the insular cortex in a homunculus-like representation of the body [55]. The insula receives inputs from the sympathetic and parasympathetic systems and from pain pathways, transmitting them to other cortical areas. The afferent information is anatomically structured, and proceeds in their integration and stabilization, from the posterior to the anterior insula [56]. The integration of this information contributes to the homeostatic response, and it provides insight into the role of the anterior insula with regard to awareness [57].

The insula is also connected to the anterior cingulate cortex (ACC) and the amygdala. The first is an important cortical area connected to the prefrontal cortex, and it is involved with consciousness and behavior. The second brain area is also connected to ACC and is responsible for fear responses [58].

The interaction between the insula, ACC, and amygdala contributes to reaching homeostasis, as the activation of the insula occurs when an event (i.e., an afferent stimulus) causes a perturbation of normal homeostatic conditions [59].

The destabilization caused by the new input may be recognized as pain, which becomes the emotion that contributes to homeostasis.

## 8. Pain and Pain Matrix

Pain is usually defined as an unpleasant sensory and emotional experience associated with actual or potential tissue damage [60]. At the same time, it involves cognitive and affective dimensions that modulate the perception and expression of pain [61]. On the other hand, consciousness is a multifaceted phenomenon encompassing different levels and aspects of awareness, such as wakefulness, attention, self-awareness, and declarative memory [62].

The interaction between pain and consciousness has been explored to understand how pain becomes a conscious sensation, how consciousness modulates pain perception and behavior, and how pain affects consciousness in different states and disorders.

Pain and consciousness are correlated via the coordinated activation of multiple brain areas, commonly described as a “pain matrix”. This is not a fixed arrangement of structures but rather a fluid system composed of several interacting networks that process different aspects of pain.

The Pain Matrix involves two main subsystems: the Lateral Neuronal Network (LNN) and the Medial Network (MN). The LNN encompasses the S2 cortex (suprasylvian parietal operculum), lateral thalamus, and the posterior insula [63] to encode the sensory discriminative information. The MN encompasses the anterior cingulate cortex (ACC), with the prefrontal cortex encoding affective–cognitive information [58]. Most of the spinothalamic afferent input reaches these areas, which are activated through direct thalamocortical connections [64], ensuring the bodily characteristics of physical pain.

In humans, the pain matrix responds consistently to noxious mechanical or thermal stimuli [65,66,67], with the LNN deputed to coding the intensity and localization of pain inputs, and the MN works with the attentional (orienting and arousing) components of pain [64,65,68,69].

Moreover, the cerebellum [70] and motor areas (e.g., the striatum, cerebellum, and the supplementary motor area) [71] are involved in pain perception and processing.

The pain experience can be further modulated by internal states, including personal beliefs, emotions, and expectations, involving a “third-order” of regions (i.e., anterolateral and orbitofrontal, ventral tegmental, and perigenual/limbic networks) linked to high-level cognition affect, and motivation [72,73].

Pain is therefore not only the result of activity in sensory or associative brain areas. The continuous interaction of the cited networks allows encoding and integration in the memory system, with regard to painful experiences [9,74,75].

## 9. Pain in DOC

**Figure 1:**
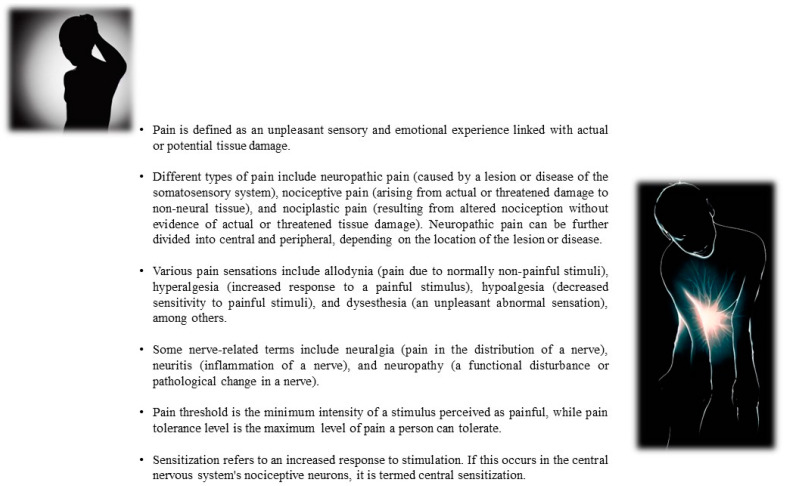
Overview of various terminologies related to pain.

Pain is a subjective experience, and its definition may vary from person to person. The International Association for the Study of Pain (IASP) approved a definition of pain in 1979, encompassing both the sensory and emotional dimensions of the experience and the association between tissue injury and pain [76]. The IASP modified its basic pain terminology in 2007, introducing new terms to describe the various aspects of pain [77]. However, the subjective nature of pain remains a fundamental aspect of the experience, and reporting on it becomes crucial, with the narrative approach being recommended to assess pain in subjects who can communicate (Figure 1, Table 2). Considering the current definition of pain, assessing it in non-communicative patients remains challenging [78].

Disorders of Consciousness (DOC) encompass a range of pathologies that affect a patient’s ability to interact with their surroundings, resulting from both traumatic and non-traumatic causes. Consciousness is generally defined as the brain’s capacity to perceive oneself and the environment, which requires adequate arousal (wakefulness) and awareness of content (sensory, cognitive, and affective experiences) [79,80]. These components are referred to as the level and content of consciousness.

Following an acquired brain injury, two possible conditions may arise: Vegetative State/Unresponsive Wakefulness Syndrome (VS/UWS) or Minimally Conscious State (MCS). VS/UWS is characterized by spontaneous eye-opening and an absence of consciousness, with only residual reflexive responses to external stimuli [81]. On the other hand, MCS presents minimal yet discernible non-reflex behaviors in response to various stimuli, although these responses may be inconsistent [81].

Clinical assessments of these conditions rely on consensus and behavioral scales, such as the Coma Recovery Scale–Revised (CRS-R), to determine the severity and extent of the disorder [81,82].

Pain can be present during both the acute phase and the subsequent intensive rehabilitation period in patients with brain injuries [83]. This may result from various factors such as skin lesions, surgical wounds, neuropathic pain, and injuries of different types. Additionally, pain may arise from nursing maneuvers and devices used during hospitalization. During the rehabilitation and chronic phases, pain can be caused by peripheral nerve lesions, central pain, spasticity, joint limitations, bedsores, paraosteoarthropathy, constipation, and post-traumatic headaches [84,85]. Central nervous system damage may also lead to chronic pain, such as thalamic pain [86,87,88].

These conditions can lead to changes in pain processing in the central nervous system and to Complex Regional Pain Syndrome (CPRS), a neuropathic pain disorder characterized by various clinical features [89]. The underlying mechanism of CPRS is multifactorial, involving abnormal neuronal transmission, autonomic dysregulation, and central sensitization. The pro-inflammatory and immunological response further contributes to peripheral sensitization and alteration of the sympathetic nervous system [89].

Painful symptoms may interfere with rehabilitation processes, limiting or delaying their effectiveness [83]. Thus, it is crucial to implement appropriate early interventions to prevent secondary damage and pain-related functional limitations, such as bedsores or muscle–tendon retraction.

## 10. Pain Treatment in DOC

There is no consensus on the appropriate pharmacological treatment of pain in patients with DOC [90]. Medication should typically be given when there are clear behavioral indications of pain. Precise dosing of pharmacotherapy is crucial to prevent interference with the evaluation and therapy strategy for recovering consciousness.

Moreover, if the strategy’s efficiency is still debatable, and yet to be proven through large-scale studies, the World Health Organization (WHO) proposes the WHO analgesic ladder, a pain management strategy developed in 1986 to provide adequate pain relief for cancer patients [91]. The ladder consists of three steps, with each step providing increasing levels of pain management options. The first step is for mild pain and involves the use of non-opioid analgesics such as NSAIDs or acetaminophen with or without adjuvants. The second step is for moderate pain and involves the use of weak opioids such as hydrocodone, codeine, or tramadol, with or without non-opioid analgesics and with or without adjuvants. The third step is for severe and persistent pain and involves the use of potent opioids such as morphine, methadone, fentanyl, oxycodone, buprenorphine, tapentadol, hydromorphone, or oxymorphone, with or without non-opioid analgesics and with or without adjuvants.

It is essential to consider that inadequate pain control may impair intentional behavioral responses, whereas excessive treatment, using high doses of opioids to decrease pain, could negatively interfere with arousal [92] and may hinder cognitive recovery and attention [90,93]. The optimal drug dosage could preserve the patient’s arousal and consciousness, reducing the risk of misdiagnosis [94,95]. Different approaches are suggested in the presence of suspected symptomatic, mild, moderate, or neuropathic pain. In the case of managing pain with symptoms, the principles of proportionality and gradualness are considered, given their interactions with current therapies. In this case, treatment approaches typically involve the use of aspirin, paracetamol, nonsteroidal anti-inflammatory drugs, opioids, and γ-aminobutyric acid (GABA)-ergic agents [90,96]. In cases of suspected mild pain, administering aspirin, paracetamol, or nonsteroidal anti-inflammatory drugs is suggested [97]. For moderate or neuropathic pain, it is recommended to use high-dose aspirin or paracetamol, oral NSAIDs, and GABAergic agents [83,90,98,99]. Finally, for suspected severe pain the use of mixed agonists/antagonists, partial agonist opioids, parenteral opioids, antidepressants, anticonvulsants, and atypical agents is usually suggested [83,90,100,101].

Since around 89% of DOC patients are characterized by spasticity [102], which is associated with pain and other symptoms (i.e., increased hypertonia, altered sensorimotor control, and muscle spasms) [103], in cases of focal spasticity, or to treat severe or worsening cases, infiltration of botulinum [104,105] is suggested. For dystonia and diffuse spasticity, improvements were instead observed by administering intrathecal baclofen [106].

## 11. Pain and Consciousness in DOC

Pain treatment is a relevant aspect of the management of DOC patients. However, pain characteristics related to the presence/absence of behavioral responses, and the modifications observed in biomarkers during noxious stimuli, can provide information on the covert content of consciousness (Table 3).

The Nociception Coma Scale (NCS) has been developed to assess pain in DOC patients [107]. The pressure of a fingernail on four limbs provides nociceptive stimulus, applied generally using an algometer, to quantify the necessary pressure to observe the behavioral response to the stimulus. The NCS consists of four subscales assessing motor, verbal, visual responses, and facial expression, allowing distinctions between reflexes (e.g., groaning or oral reflex movements) and higher-level behaviors (e.g., pain localization and crying or intelligible verbalization). Since the visual subscale does not show significant changes between a noxious and a non-noxious condition, NCS was recently substituted by its revised version (NCS-R) [108]. The absence of the visual subscale does not alter its sensitivity, maintaining the same clinometric property of the NCS [109], with higher total scores in MCS than in VS/UWS patients.

In a study on 64 patients, Chatelle and colleagues [108] observed that the total scores and subscores (motor, verbal, and facial) of the NCS were higher in noxious than non-noxious stimulation conditions. They identified an NCS cut-off value of four that distinguished the patients who received a noxious stimulation from those who received a non-noxious stimulation.

A successive neuroimaging study in DOC with fluorodeoxyglucose (FDG)-PET showed positive correlations between brain activity from the ACC and NCS-R scores, indicating a correlation with pain processing [110]. Considering the NCS-R, Chatelle and colleagues proposed a cut-off value of two to differentiate nociception from pain [111]. However, in a retrospective study on the neural basis for pain experience based on the preservation of brain metabolism as assessed by FDG-PET, Bonin and colleagues, suggested a conservative NCS-R cut-off score of less than five to identify pain in these patients [112].

Concerning the modality with which to administer the nociceptive stimulus, Formisano et al. [113] proposed the use of NCS(-R) with personalized stimulations (for example, opening the hand, abducting the upper limbs, and mobilizing the head), which may cause different reactions compared with simpler pressure applied to the fingernail bed.

Again, a multicentric study involving 40 healthy volunteers and 60 DOC patients found that VS/UWS and MCS patients had lower pressure pain thresholds than healthy participants, suggesting further research on possible pain hypersensitivity in patients with severe brain injuries and multiple co-morbidities is needed [114].

In a recent study involving 70 VS/UWS patients, Cortese and colleagues [115] provided evidence to show that an accurate assessment of pain could predict changes in consciousness level with an accuracy of 84% when using the NCS, and 72% when adopting the NCS-R. The results indicated that a change in behavioral response, following a nociceptive stimulation, with a total score for the NCS of ≥5 and ≥3 for the NCS-R, can predict positive outcomes with regard to the condition of MCS.

However, it is crucial to consider the patient’s clinical condition in the pain assessment. DOC patients could have developed severe spasticity or they may have been intubated, making pain assessment more complex [102,116]. In patients with tracheostomy, it is necessary to consider lower cut-off values to distinguish nociception from pain because of lower verbal sub-scares in these patients [117].

Interestingly, Cortese and colleagues [118] showed the possibility of observing trace conditioning in VS/UWS patients without any behavioral responses to nociceptive stimuli. The study measured the Galvanic Skin Response (GRS) and Heart Rate Variability (HRV) to assess responses to nociceptive stimuli in 13 healthy subjects and 37 VS/UWS patients. Eight VS/UWS, which all showed trace conditioning to the noxious stimulus, were diagnosed as MCS within one month.

In the context of autonomic responses to pain, Heart Rate Variability (HRV) emerges as a valuable marker for assessing nociceptive responses in cases of experimentally induced pain. Enhanced parasympathetic activity has been associated with better self-regulation capacities and increased pain inhibition capacity [119]. In a study conducted by Tobalbini and colleagues, involving 24 patients diagnosed with disorders of consciousness (DOC), it was observed that nociceptive stimuli could lead to changes in autonomic function. This change was characterized by increased sympathetic activity and reduced vagal modulation [120]. In two separate studies—one involving 21 DOC patients, including 11 with UWS/VS [121], and the other involving 24 DOC patients, including 12 with UWS/VS [120]—a reduction in cardiac complexity (i.e., HRV entropy) was noted in UWS/VS patients during exposure to noxious stimuli. Additionally, in an EEG-based study involving 21 DOC patients, pain stimulation was linked with a higher parietal response in the delta frequency band, lower activation in the left frontal region, and increased Galvanic Skin Response (GSR) and Heart Rate [122].

## 12. Conclusions

Pain is a complex and subjective experience that involves various brain networks. Assessing pain in patients with disorders of consciousness is particularly challenging, and clinicians must rely on careful observations and behavioral scales to determine the presence and intensity of pain.

As a multidimensional experience, pain is distinct from nociception and involves salience and arousal responses throughout the body and in overlapping brain circuits. An analysis of autonomic nervous system activity was suggested as a surrogate measure of salience and arousal to improve the accuracy and specificity of functional neuroimaging analyses and help to overcome current difficulties in assessing pain-specific responses in the brain [123].

Pain has well-established effects on attention. Research has shown that the effects of acute and chronic pain on attention are different, suggesting that different models need to be developed to understand these phenomena [124].

The aversive experience called “pain” results from the coordinated activation of multiple brain areas (pain matrix), which progresses rapidly to the recruitment of anterior insular and fronto-parietal networks, and finally, to the activation of the perigenual, posterior cingulate, and hippocampal structures. During the initial second following a stimulus, the interaction between sensory and high-level networks intensifies, potentially playing a key role in access to consciousness [125]. A possible model suggests that various brain networks facilitate pain processing transitions from unconscious sensorimotor and limbic reactions to conscious awareness. This process, which integrates personal memories and self-awareness, occurs only during alert states. However, even unconscious individuals may form implicit memories from repeated pain exposure [125].

Recognizing pain, as opposed to just a nociceptive response, in patients who are unable to communicate it is of great significance. Pain is a distressing experience that can negatively impact a patient’s quality of life. Identifying and managing pain can provide significant relief and improve overall patient well-being. Pain responses can provide valuable insights into a patient’s condition. In patients with disorders of consciousness, the presence of pain might indicate residual or covert consciousness, influencing prognostic considerations and care plans.

The ethical considerations involved in this context are also substantial [126]. In DOC patients, difficulties in accurately predicting an individual’s ability to experience pain and distress pose significant obstacles when trying to offer sufficient pain relief, even when employing verified pain assessment tools [127].

The appropriate pharmacological treatment of pain in these patients is still under debate [90]. Clinicians should consider the potential side effects of medication and its impact on consciousness and cognitive recovery when determining the appropriate pharmacological treatment of pain in DOC patients.

The NCS and NCS-R are the behavioral scales used to assess pain in DOC patients, providing subscales that allow for disentangling reflexes from higher-level behaviors, thus allowing differentiation between nociception and pain [107,108]. Additionally, personalized stimulation protocols that induce different responses may be used to enhance pain assessment accuracy [113]. Recent studies have shown that an accurate pain assessment might predict changes in the level of consciousness in DOC patients with a high degree of accuracy [115]. Furthermore, the presence or absence of behavioral responses to nociceptive stimuli and modifications observed in biomarkers can help differentiate nociception from pain and identify covert content of consciousness in patients diagnosed as VS/UWS [118].

Pain research on DOC provides evidence to suggest the significant need to optimize pharmacological treatments that consider the cognitive recovery of these patients, alongside investigating the long-term effects of repeated pain exposure.

It is advisable to adopt a multidisciplinary approach to ensure effective pain management and optimize the recovery process in patients with DOC. Accurately assessing pain responses can provide insights into changes in the individual’s level of consciousness, further emphasizing the ongoing importance of managing pain in these patients.

Further research could delve into novel neuroimaging techniques, like high-resolution functional Magnetic Resonance Imaging (fMRI) [128,129], and innovative non-invasive neuromodulation methods, such as transcranial direct current stimulation (tDCS) [130] or transcranial magnetic stimulation (TMS) [131,132]. These tools can provide more detailed insights into the brain’s response to pain stimuli in DOC patients. Adopting machine learning algorithms and advanced computational models could also enable the development of more reliable and specific pain biomarkers that may distinguish between nociception and pain perception [133,134]. Moreover, studies that aim to understand the effects of repeated nociceptive stimuli over time, particularly concerning the formation of implicit memories in unconscious individuals, could help establish if, and to what extent, repeated pain exposure influences a patient’s recovery trajectory and their transition from unconscious to conscious states. Understanding the ethical implications of such studies will also be crucial. Ultimately, integrating these diverse research directions can help to establish a more comprehensive framework for assessing and managing pain in DOC patients, and it may unveil the hidden facets of consciousness, significantly advancing our understanding of the human brain and consciousness.

## Figures and Tables

**Table 1:** Nerve Fiber Characteristics and Associated Pain.

Nerve Fiber	Conduction Speed	Diameter	Myelination	Type of Pain	Pain Characteristics
A Delta	Fast (5–30 m/s)	Medium (2–5 μm)	Myelinated	Acute pain	Sharp, pricking, or stabbing pain is often associated with acute injuries. Felt immediately following an injury and is usually localized to the area of injury. It can be described as “fast pain.”
A Beta	Very fast (33–75 m/s)	Large (5–20 μm)	Myelinated	Typically not associated with pain	Generally responsible for transmitting non-painful stimuli such as touch, pressure, and proprioception. However, in nerve injury or disease cases, they may become involved in transmitting pain signals.
C Fibers	Slow (0.5–2 m/s)	Small (0.4–1.2 μm)	Unmyelinated	Chronic pain	Dull, throbbing, aching, or burning pain is typically associated with chronic conditions. Felt a short while after an injury and can be widespread. It can be described as “slow pain”.

**Table 2:** Classification and Description of Pain Types and Associated Terminology.

Term	Definition of Pain
Pain	An unpleasant sensory and emotional experience associated with actual or potential tissue damage or described in terms of such damage.
Neuropathic Pain	Pain caused by a lesion or disease of the somatosensory system.
Central Neuropathic Pain	Neuropathic pain resulting from a lesion or disease of the central somatosensory nervous system.
Peripheral Neuropathic Pain	Neuropathic pain resulting from a lesion or disease of the peripheral somatosensory nervous system.
Nociceptive Pain	Pain that arises from actual or threatened damage to non-neural tissue and is due to the activation of nociceptors.
Nociplastic Pain	Pain that arises from altered nociception despite no clear evidence of actual or threatened tissue damage.
	**Types of Pain Sensations:**
Allodynia	Pain due to a stimulus that does not normally provoke pain.
Hyperalgesia	An increased response to a stimulus which is normally painful.
Hypoalgesia	Decreased sensitivity to painful stimuli.
Anesthesia dolorosa	Pain in an area or region which is anesthetic.
Dysesthesia	An unpleasant abnormal sensation, whether spontaneous or evoked.
Hyperesthesia	Increased sensitivity to stimulation, excluding the special senses.
Hyperpathia	A painful syndrome characterized by an abnormally painful reaction to a stimulus, especially a repetitive stimulus, as well as an increased threshold.
Hypoesthesia	Decreased sensitivity to stimulation, excluding the special senses.
Paresthesia	An abnormal sensation, whether spontaneous or evoked.
	**Nerve-Related Terms:**
Neuralgia	Pain in the distribution of a nerve or nerves.
Neuritis	Inflammation of a nerve or nerves.
Neuropathy	A disturbance of function or pathological change in a nerve.
Nociception	The neural process of encoding noxious stimuli.
Nociceptive Neuron	A neuron that is capable of detecting noxious stimuli.
Nociceptive Stimulus	A stimulus that is damaging or threatens damage to normal tissues.
Nociceptor	A receptor preferentially sensitive to a noxious stimulus or to a stimulus which would become noxious if prolonged.
Noxious Stimulus	A stimulus that is damaging to normal tissues.
	**Pain Threshold and Tolerance:**
Pain Threshold	The minimum intensity of a stimulus that is perceived as painful.
Pain Tolerance Level	The maximum level of pain which a subject is prepared to tolerate.
	**Sensitization**:
Sensitization	An increased response to stimulation.
Central Sensitization	Increased responsiveness of nociceptive neurons in the central nervous system to their normal or subthreshold afferent input.

**Table 3:** Common Signs and Characteristics Evaluated in Pain in DOC Patients.

Signs/Symptoms/Characteristics	Description	Evaluation in MCS Patients	Evaluation in VS/UWS Patients
Motor Response	Assessed in the NCS and NCS-R as part of the behavioral response to pain stimuli.	Higher-level responses, such as flexion or withdrawal.	Lower-level responses, such as abnormal posturing or none/flaccid.
Verbal Response	Evaluated in the NCS and NCS-R; factors such as crying, groaning, or intelligible verbalization are considered.	Higher-level responses, such as vocalization or intelligible verbalization.	Lower-level responses, such as groaning or no response. Necessary to consider lower responses due to tracheostomy conditions.
Facial Expression	Assessed as part of NCS and NCS-R, includes evaluation of reactions like grimacing.	More expressive, such as a cry or grimace.	Startled/oral reflexive movements or no response.
Visual Expression	Assessed as part of NCS, includes evaluation of reactions like fixation.	Higher-level responses, such as fixation and eye movements.	Startled or no response.
Pain Localization	Higher-level behavior indicative of pain as assessed by the NCS and NCS-R.	Observable.	Not observable.
Personalized Stimulation Reaction	Involves reactions to personalized stimuli, such as opening the hand, abducting the upper limbs, and mobilizing the head.	More demonstrated.	Less demonstrated.
Cardiac Frequency (Heart Rate Variability)	HRV can be used to assess autonomic responses to pain, such as increased sympathetic activity and reduced vagal modulation.	More stable HRV.	Increased sympathetic activity and reduced vagal modulation.
Galvanic Skin Response (GSR)	GSR measurements can indicate physiological responses to pain stimuli.	Trace conditioning was observed in healthy controls. No studies are present for MCS patients.	Can show trace conditioning to noxious stimuli.
Tracheostomy Condition	Pain assessment should consider lower cut-off values for tracheostomized patients due to lower verbal subscale scores.	Not applicable.	Lower cut-off values for nociception.
Spasticity	Severe spasticity can affect pain assessment in DOC patients.	Possible.	Possible.
